# Inhibitory effect of S-nitroso-N-acetylpenicillamine on the basolateral 10-pS Cl^-^ channel in thick ascending limb

**DOI:** 10.1371/journal.pone.0284707

**Published:** 2023-04-21

**Authors:** Shiwei Ye, Peng Wu, Zhongxiuzi Gao, Mingyan Wang, Li Zhou, Zhi Qi

**Affiliations:** 1 Department of Basic Medical Sciences, School of Medicine, Xiamen University, Xiamen, China; 2 Department of Nephrology, The First Affiliated Hospital of Zhengzhou University, Zhengzhou, China; Southern University and A&M College, UNITED STATES

## Abstract

We have previously reported that L-arginine, a nitric oxide synthase substrate, inhibits the basolateral 10-pS Cl^-^ channel through the cGMP/PKG signaling pathway in the thick ascending limb (TAL). As a NO releasing agent, the effect of *S*-nitroso-*N*-acetyl-penicillamine (SNAP) on the channel activity was examined in thick ascending limb of C57BL/6 mice in the present study. SNAP inhibited the basolateral 10-pS Cl^-^ channel in a dose-dependent manner with an IC50 value of 6.6 μM. The inhibitory effect of SNAP was abolished not only by NO scavenger (carboxy-PTIO) but also by blockers of soluble guanylate cyclase (ODQ or LY-83583), indicating that the cGMP-dependent signaling pathway is involved. Moreover, the inhibitory effect of SNAP on the channel was strongly attenuated by a protein kinase G (PKG)-specific inhibitor, KT-5823, but not by the PDE2 inhibitor, BAY-60-7550. We concluded that SNAP inhibited the basolateral 10-pS Cl^-^ channels in the TAL through a cGMP/PKG signaling pathway. As the 10-pS Cl^-^ channel is important for regulation of NaCl absorption along the nephron, these data suggest that SNAP might be served as a regulator to prevent high-salt absorption related diseases, such as hypertension.

## Introduction

Hypertension is influenced by multiple risk factors, among which high NaCl intake is one of the risk factors that has been studied the most. It is believed that increased NaCl intake elevates blood pressure and thus favoring the development of hypertension [1]. Considerable evidence indicated that it is the combination of Na^+^ and Cl^-^, rather than Na^+^ per se, is responsible for the development of salt-sensitive hypertension [1–5]. For example, in Dahl rats (an animal model of salt-sensitive hypertensive rat), selective loading with only Na^+^ or Cl^−^ but not both failed to induce changes in blood pressure [2,6,7]. It has been indicated that decrease in absorption of Cl^−^ in renal tubular might be the reason for why sodium salt in the absence of chloride could not be able to increase blood pressure in the Dahl salt-sensitive rat [8]. Cl^−^ is the richest anion in both extracellular and intracellular environments of our body [9]. Therefore, it is not surprise that Cl^−^ transport along the nephron is important in the regulation of extracellular fluid volume as well as blood pressure [10,11]. These results suggest that control of not only Na^+^ absorption but also Cl^−^ absorption is critical for regulation of blood pressure and thus contributes to the development of salt-sensitive hypertension [2,9,10,12].

The thick ascending limb (TAL) of the loop of Henle reabsorbs over one-fifth of the filtered NaCl while absorbing no water. By doing so, the TAL segment helps the nephron to establish and maintain the hypertonic medullary solute gradient, generate dilute tubular fluid, and thus plays important roles in regulating fluid volume and blood pressure [13–15]. Improper regulation of Cl^−^ absorption by this segment has been implicated in salt-sensitive hypertension [14]. Cl^−^ absorption is a two-step process in the TAL [16]. Cl^−^ entries across the apical membrane via Na^+^-K^+^-2Cl^−^ co-transporter. Generally, the proper function of Na^+^-K^+^-2Cl^−^ co-transporter requires the simultaneous presence of all three ions, which means that the transport of Na^+^ and Cl^−^ across the apical membrane is dependent on each other. Once inside the cell, Cl^−^ exits across the basolateral membrane down a favorable electrochemical gradient through the basolateral Cl^−^ channels. Ion-substitution experiment showed that the basolateral Cl^−^ channels provide the major pathway for Cl^−^ exit [17]. These results suggest that Cl^−^ channels in the basolateral membrane play an important role in controlling Cl^−^ absorption in the TAL.

Several researches using patch-clamp technique have found that two different Cl^−^ channels exist in the TAL: the smaller one with conductance of ~10 pS and the larger one with conductance of ~30–40 pS. It has been demonstrated that the smaller one is the major type of the basolateral Cl^−^ channels in the TAL [18–21]. For example, our previous study has showed that the single channel activity of the 10-pS Cl^−^ could be obtained in 314 patches among the total of 1,147 patches investigated, whereas the single channel activity of the 30-pS Cl^−^ channel was observed in only 68 patches [21]. Furthermore, we have demonstrated that nitric oxide (NO) synthase substrate (L-arginine) could inhibit the basolateral 10-pS Cl^−^ channel in the TAL through the cGMP/PKG signaling pathway [21]. As a NO releasing agent, S-nitroso-N-acetyl-penicillamine (SNAP) might be a potential candidate for regulation of NaCl absorption by affecting the basolateral 10-pS Cl^−^ channel activity. In the present study, we studied the detailed molecular mechanism of SNAP on the regulation of this 10-pS Cl^−^ channel.

## Materials and methods

### Preparation of the TAL for single channel recordings

The pathogen-free C57BL/6 mice (male, 5 wks old) were from Laboratory Animal Center of Xiamen University (Xiamen, China). All animal procedures were in strict accordance with the National Institutes of Health’s Guidelines for the Care and Use of Laboratory Animals. All the experimental protocols were approved by the Animal Care and Use Committee of Xiamen University following the Guide for the Care and Use of Laboratory Animals [22]. The mice had free access to water and were fed with a control diet. Every effort was made to minimize the number of animals used and to minimize suffering. After the mice had been euthanized by pentobarbital administration (150 mg/kg) followed by cervical dislocation, the kidneys were removed immediately and thin coronal sections (1-mm) were cut with a razor blade. The TALs were dissected in a HEPES-buffered NaCl solution containing 1 mg/mL collagenase type 1A (Sigma, St. Louis, MO) at 37°C for 55 minutes. Then, the dissected TAL was transferred onto a cover glass (5 mm × 5 mm) that was coated with poly-lysine (Sigma) overnight to immobilize the tubule. The cover glass was placed in a chamber mounted on an inverted microscope (Olympus) for single channel recordings and the tubules were superfused with HEPES-buffered NaCl solution containing (mM): 5 KCl, 140 NaCl, 1.8 MgCl_2_, 1.8 CaCl_2_ and 10 HEPES with pH of 7.4.

### Single channel recordings

Single-channel recordings were obtained from cell-attached configuration of patch-clamp technique. The pipette solution contains (mM): 1.8 MgCl_2_, 140 NaCl and 10 HEPES with pH of 7.4. Single channel activity was defined as *NP*_o_, a product of channel number (*N*) and open probability (*P*_*o*_). The *NP*_o_ was calculated from single channel recordings using the following equation:

$$NP_{o}=\sum \;(1t_{1}+2t_{2}+\ldots it_{i})$$ (1)

where *t*_i_ is the fractional open time spent at each of the observed current levels. For the single channel activity, the single channel recordings with duration of 60 seconds, which were obtained at least 2 min after addition of SNAP, were analyzed.

### Chemicals

KT-5823, SNAP, carboxy-PTIO and 1H-[1,2,4]Oxadiazolo[4,3-a]quinoxalin-1-one (ODQ) were from Sigma (St. Louis, MO). LY-83583 and BAY-60-7550 were from Santa Cruz Biotechnology (Santa Cruz, CA). ODQ, LY-83583 and BAY-60-7550 were dissolved in dimethyl sulfoxide (DMSO). To ensure that the channel activity was not affected by DMSO, its final concentration in the bath was less than 0.1%.

### Statistical analysis

An IC50 value was obtained by fitting concentration dependence data from single channel recordings to the following equation:

$$I\;(\%)={\left[SNAP\right]}^{H}/(IC_{50}{}^{H}+{\left[SNAP\right]}^{H})$$ (2)

in which I (%) is the percentage of inhibition, H represents the Hill coefficient and [SNAP] represents concentration of SNAP. The percentage of inhibition at the test potential is calculated by the following equation:

$$I\;(\%)=[1‐NPo_{\left(SNAP\right)}/NPo_{\left(\mathrm{control}\right)}]\times 100$$ (3)

where *NPo*_(SNAP)_ and *NPo*_(control)_ represent *NPo* of the channels in the presence of SNAP and under control condition, respectively. Data are shown as mean ± SEM. Differences in means were tested with paired sample t-test and were accepted as significant if *P* < 0.05.

## Results

**Fig 1:**
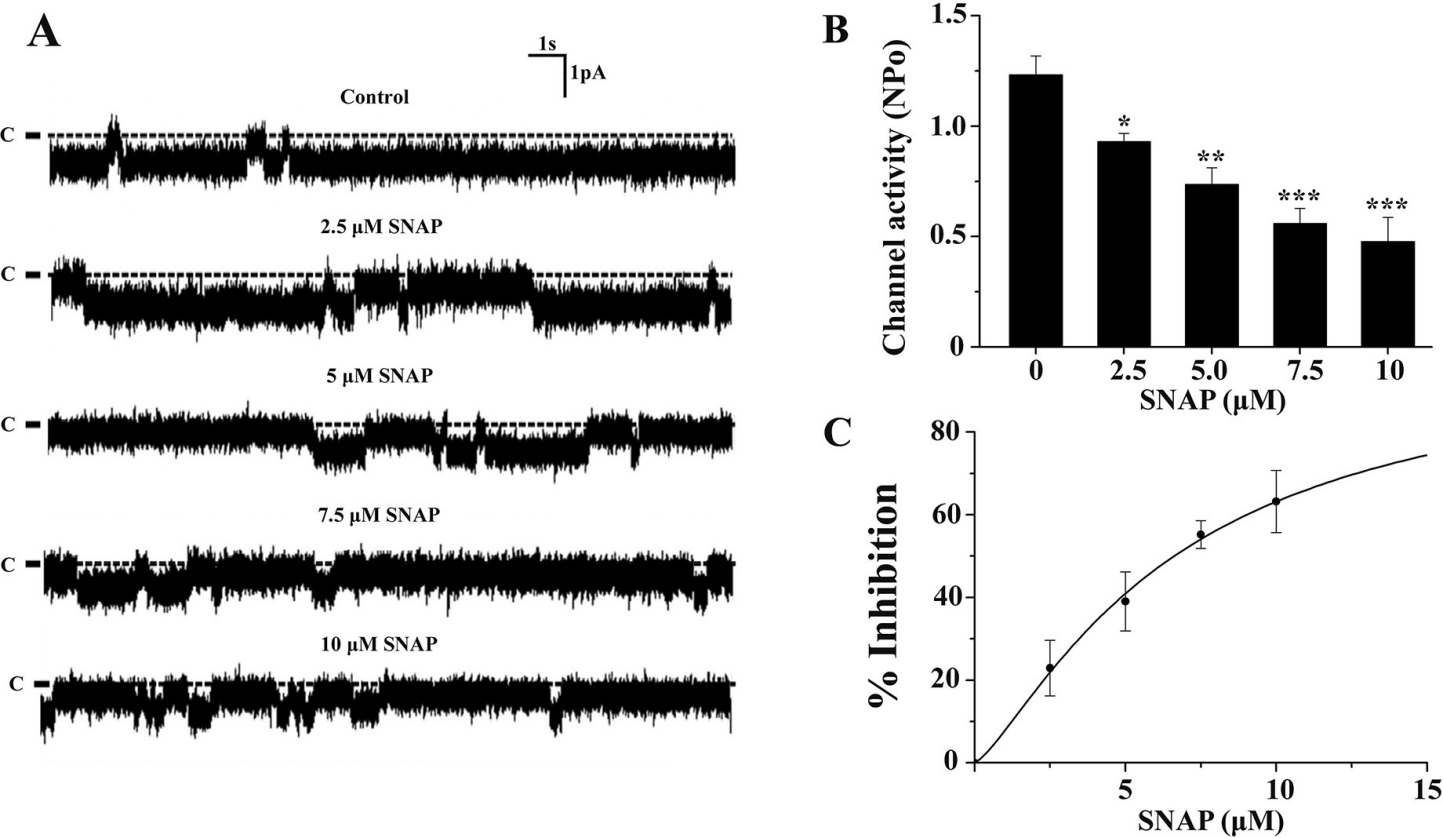
Inhibitory effect of SNAP on the basolateral 10-pS Cl^-^ channel in the TAL. (A) Representative single channel traces of the basolateral 10-pS Cl^-^ channel at a holding potential of -60 mV in the control and during successive exposure to different concentration of SNAP (2.5–10 μM) from the same patch in cell-attached configuration. The channel closed level is indicated by the dotted line and “C”. (B) Statistical summary for dose-response effect of SNAP on the 10-pS Cl^-^ channels from 5 sets of experiments. Each set of experiment was obtained by successive exposure to different concentration of SNAP (2.5–10 μM) from the same patch. Asterisk indicates the significant difference between the control (no SNAP) and SNAP treated groups. (*: *P* < 0.05, **: *P* < 0.01, ***:*P* < 0.001, paired sample t-test). (C) The curve is the best fit to percentage of inhibition (Eq 3) against the [SNAP] according to the Hill equation (Eq 2) with IC_50_ = 6.62 μM.

In most of the experiments, the *NPo* was different from different patches even under control condition. To avoid the effect of the values of different *NPo* of different patches on the experimental results, we applied different concentrations of SNAP on the same patch and then using paired sample t-test for statistical analysis. Fig 1A is a set of representative single channel recordings from the same cell-attached patch, showing that the *NPo* of the channel in the same patch was gradually decreased with the increase in [SNAP] from 0 to 10 μM. The statistical analysis on dose-response effect in Fig 1B showed that the mean *NPo* of the channel was 1.23 ± 0.08 before application of SNAP, and it decreased to 0.93 ± 0.04, 0.74 ± 0.07, 0.56 ± 0.07, and 0.48 ± 0.11 when [SNAP] was increased to 2.5, 5, 7.5 and 10 μM, respectively. A nonlinear curve fit of the Hill equation (Eq 2) to the data points in Fig 1C yielded an IC_50_ value of 6.62 ± 0.20 μM and a Hill coefficient of 1.31 ± 0.08 (n = 5), respectively. Because 5 μM SNAP was a suitable concentration to inhibit the channel, we used this concentration in the following experiments.

**Fig 2:**
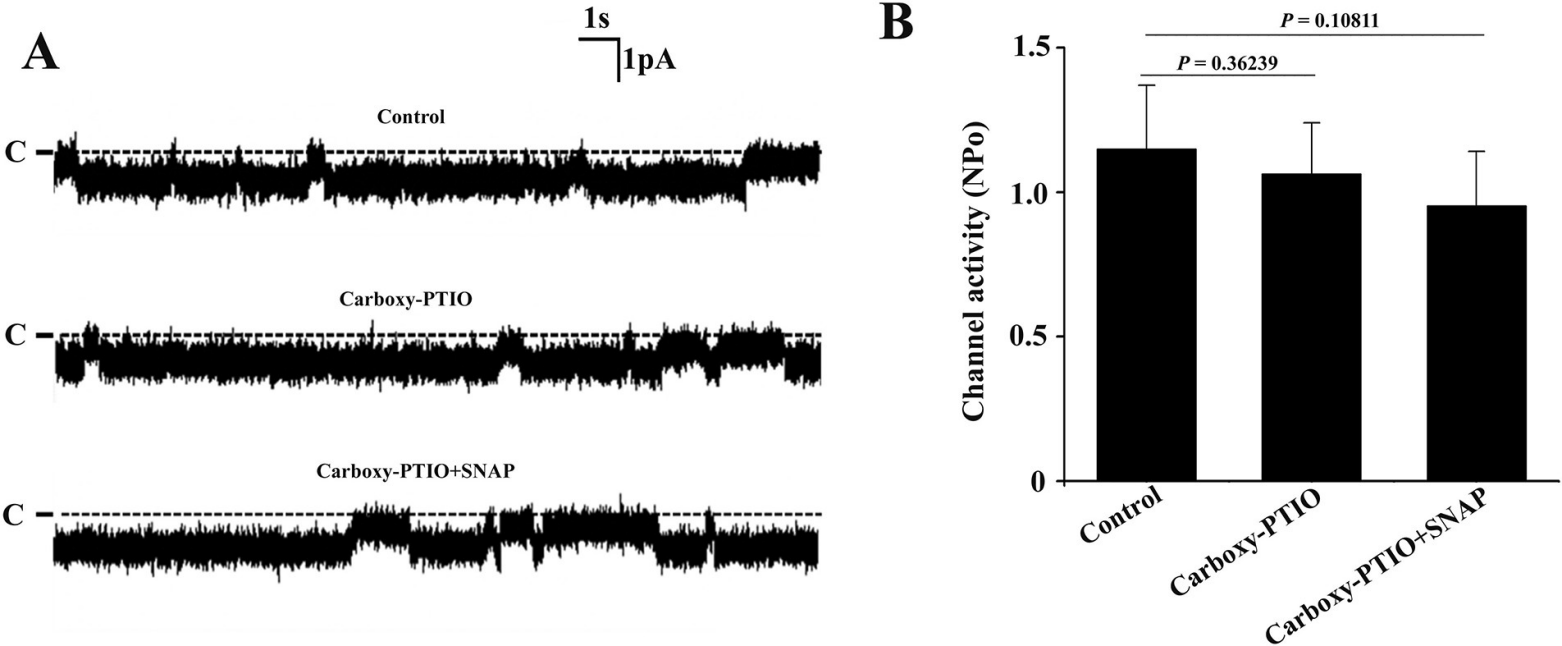
Scavenging NO diminishes the inhibitory effect of SNAP on the 10-pS Cl^-^ channels. (A) A set of singe channel recordings from the same patch in a cell-attached patch under control condition, in the presence of 10 μM carboxy-PTIO alone as well as in the presence of 10 μM carboxy-PTIO + 5 μM SNAP. The holding potential was −60 mV and the channel closed level is indicated by the dotted line and “C”. (B) Statistical summary showing that carboxy-PTIO abolishes the inhibitory effect of SNAP on the channels (n = 6).

To judge whether the inhibitory effect of SNAP on the single channel activity of the 10-pS Cl^-^ channels is due to its release of NO, we used a NO scavenger (carboxy-PTIO) to eliminate NO. Fig 2A illustrates a set of single channel recordings from the same patch under control condition, in the presence of carboxy-PTIO as well as in the presence of both carboxy-PTIO and SNAP. Application of 10 μM carboxy-PTIO alone did not affect the single channel activity. However, SNAP failed to inhibit the channel activity if carboxy-PTIO was applied. Statistical results from 6 set of experiments demonstrated that 10 μM carboxy-PTIO did not significantly affect the single channel activity, but the inhibitory effect of SNAP on the Cl^-^ channels was abolished in the presence of carboxy-PTIO (Fig 2B), suggesting that the effect of SNAP to inhibit the channel was due to its release of NO.

**Fig 3:**
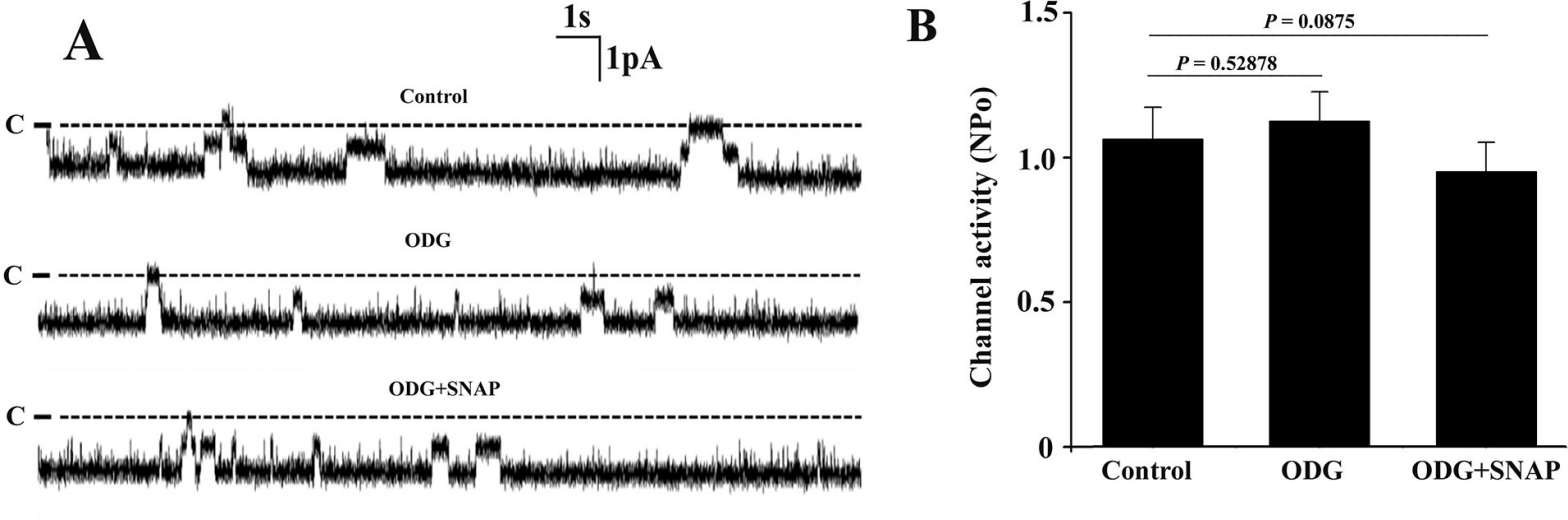
Soluble guanylyl cyclase (sGC) inhibitor ODQ blocks the inhibitory effect of SNAP on the 10-pS Cl^-^ channels in the TAL. (A) Representative single channel recordings from the same cell-attached patch under control condition, in the presence of 10 μM ODQ alone as well as in the presence of both ODQ (10 μM) and SNAP (5 μM). The holding potential was −60 mV and the channel closed level is indicated by the dotted line and “C”. (B) The bar graph showing that ODG abolishes the inhibitory effect of SNAP on the channels (n = 5).

**Fig 4:**
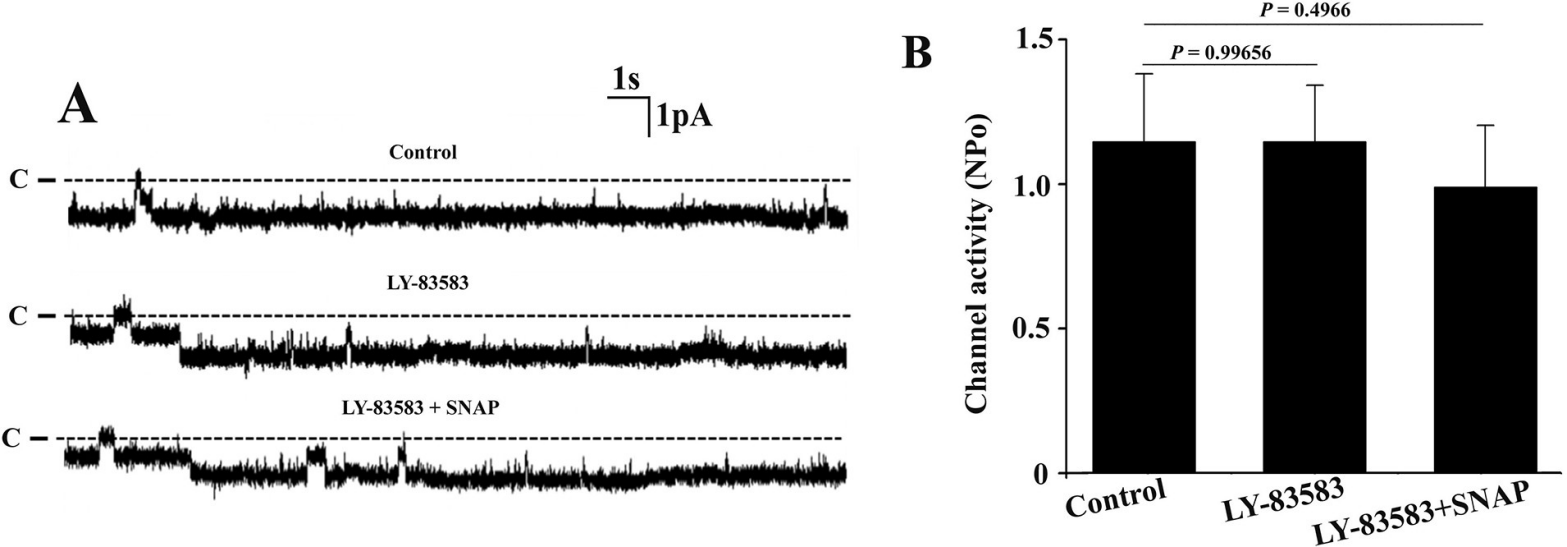
Soluble guanylyl cyclase inhibitor LY-83583 prevents the effect of SNAP on the 10-pS Cl^-^ channels. (A) Representative single channel recordings from the same cell-attached patch under control condition, in the presence of 10 μM LY-83583 alone as well as in the presence of both LY-83583 (10 μM) and SNAP (5 μM). The holding potential was -60 mV and the channel closed level is indicated by the dotted line and “C”. (B) The bar graph showing that LY-83583 eliminates the inhibitory effect of SNAP on the channel.

To investigate whether soluble guanylate cyclase (sGC) was involved in the inhibitory effect of SNAP on the channel activity, ODQ, a sGC inhibitor, was used in the following experiments. Fig 3A is a set of single channel recordings from the same patch under control condition, in the presence of sGC blocker ODG (10 μM) as well as in the presence of both ODG (10 μM) and SNAP (5 μM). Statistical results from 5 set of experiments demonstrate that ODG alone did not affect the single channel activity. However, application of ODG blocked the inhibitory effect of SNAP on the channels. To further confirm this result, we examined whether LY-83583, another specific sGC blocker, could also prevent the effect of SNAP on the channel. Fig 4A is a set of single channel recording from the same patch showing that even though 10 μM LY-83583 alone did not affect the channel activity, it could prevent the inhibitory effect of SNAP on the channels. Statistical analysis from 5 sets of experiments demonstrate that application of SNAP did not significantly inhibit the Cl^−^channel activity in the TAL that was pre-treated with LY-83583 (Fig 4B). These results suggest that the inhibitory effect of SNAP on the channel is due to its activation of sGC.

**Fig 5:**
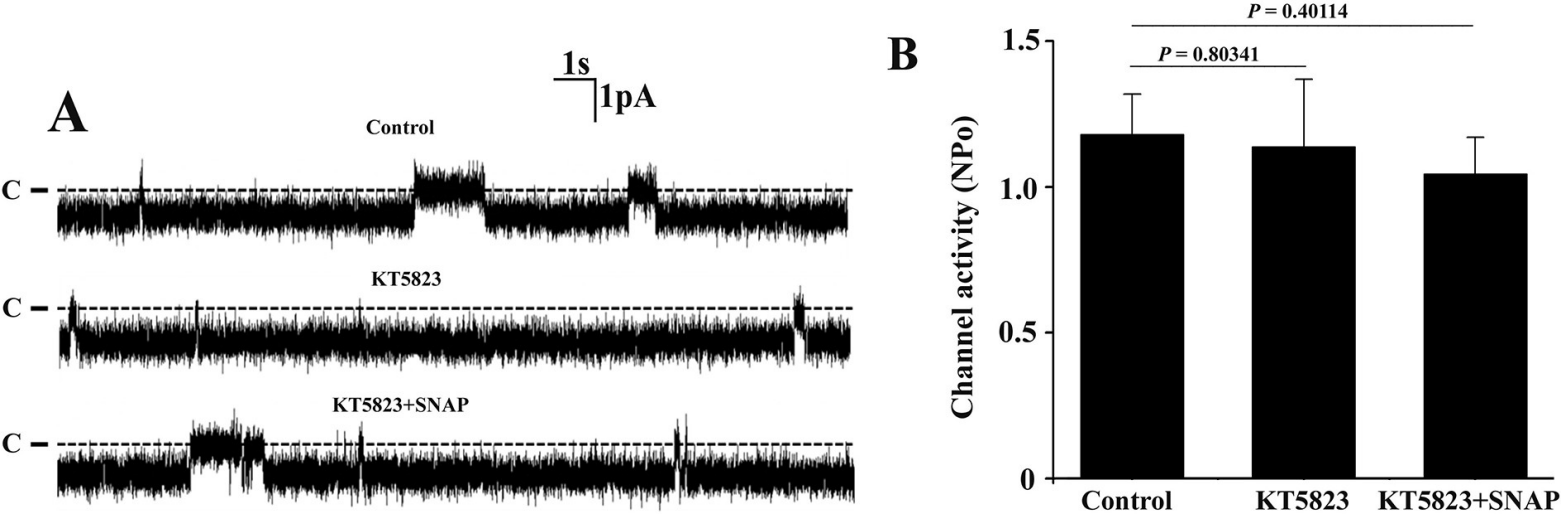
Inhibition of PKG abolishes the effect of SNAP on the 10-pS Cl^-^ channels in the TAL. (A) Representative single channel recordings from the same cell-attached patch under control condition, in the presence of 10 μM KT5823 alone as well as in the presence of both KT5823 (10 μM) and SNAP (5 μM). The holding potential was -60 mV and the channel closed level is indicated by the dotted line and “C”. (B) The bar graph showing that KT5823 eliminates the inhibitory effect of SNAP on the channel.

To examine whether cGMP-dependent protein kinase (PKG) signaling pathway is involved, the effect of SNAP on the 10-pS Cl^−^ channels was investigated in the TAL pretreated with PKG inhibitor KT-5823. A set of single channel recordings from the same patch (Fig 5A) and statistical summary (Fig 5B) showed that KT-5823 (5 μM) alone had no significant effect on the single channel activity of the 10-pS Cl^−^ channel. However, inhibition of PKG could eliminate the effect of SNAP on the channel activity after the TAL was pretreated with KT-5823.

**Fig 6:**
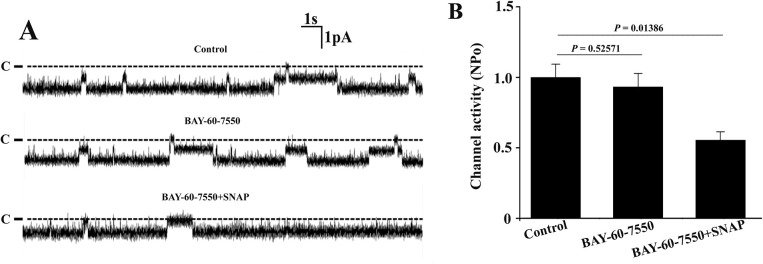
Inhibition of PDE2 fails to abolish the effect of SNAP on the 10-pS Cl^-^ channels in the TAL. (A) Representative single channel recordings from the same cell-attached patch under control condition, in the presence of 10 μM BAY-60-7550 alone as well as in the presence of both BAY-60-7550 (10 μM) and SNAP (5 μM). The holding potential was -60 mV and the channel closed level is indicated by the dotted line and “C”. (B) The bar graph showing that BAY-60-7550 plays no role in the inhibitory effect of SNAP on the 10-pS Cl^-^ channels.

Finally, to test whether the cGMP-stimulated phosphodiesterase II (PDE2) is involved, BAY 60–7550, a specific PDE2 inhibitor was used in the following experiment. A set of single channel recordings from the same patch (Fig 6A) and statistical summary (Fig 6B) showed that BAY-60-7550 alone did not significantly affect the 10-pS Cl^−^ channel activity. However, SNAP has the similar inhibitory effect on the channel even in the presence of BAY-60-7550, suggesting that PDE2 is not involved in mediating the inhibitory effect of SNAP on the single channel activity of the 10-pS Cl^-^ channel.

## Discussion

The basolateral Cl^-^ channels of the TAL belong to the ClC family. Two family members of ClC are found in the TAL: ClC-K1 and ClC-K2, corresponding to CLC-NKA and CLC-NKB in humans. For a functional channel, barttin subunit is necessary to facilitate the insertion of the channel into the plasma membrane of the cell [23,24]. Although both ClC-K1 and ClC-K2 are expressed in the TAL, several lines of evidence indicate that ClC-K2 is the basolateral 10-pS channel [13]; 1) Only ClC-K2 can be observed by immunohistochemistry technique in the basolateral membrane of the TAL [25,26]; 2) Judged by electrophysiological properties, especially ion selectivity of the channel, it has been suggested that CLC-K2 is the dominant Cl^−^ channel in the TAL [27]; 3) Single channel recordings indicated that the 10-pS channel is dominant Cl^-^ channel in the TAL [18,21]. 4) Electrophysiological study on knockout mice has showed that ClC-K2 is the 10-pS Cl^-^ channel and it accounts for most of the basolateral Cl^-^ current in the TAL [13].

Furthermore, as the major pathway for Cl^-^ exits across the basolateral membrane [26,28], this 10-pS ClC-K2 channel has been suggested to be essential for NaCl absorption in the TAL, which in turn plays roles in salt-sensitive increases in extracellular fluid volume and blood pressure regulation [13,29]. Mutations that lead to loss-of-function of the channel cause Bartter’s syndrome in humans indicates that ClC-Kb is responsible for NaCl absorption in the TAL [30–32]. Importantly, overexpression of ClC-K2 in Dahl rats increased Cl^-^ channel activity, which might contribute to the elevation of blood pressure of the rats [33]. It has been found that T481S mutation in ClC-Kb strongly increased the Cl- conductance by a factor of 20 [34]. Interestingly, there is a strong association between the ClC-Kb^T481S^ carriers and higher average blood pressure as well as fraction of participants who had hypertensive blood pressure levels, suggesting that the mutation ClC-Kb^T481S^ of the channel may predispose to the development of hypertension [35,36]. Electrophysiological studies performed on knockout mice indicate that ClC-K2 is essential for salt absorption in the TAL [13]. Taken together, these results suggest that increase in salt absorption by activation of the basolateral 10-pS Cl^-^ channel (ClC-K2) in the TAL is associated with hypertension.

**Fig 7:**
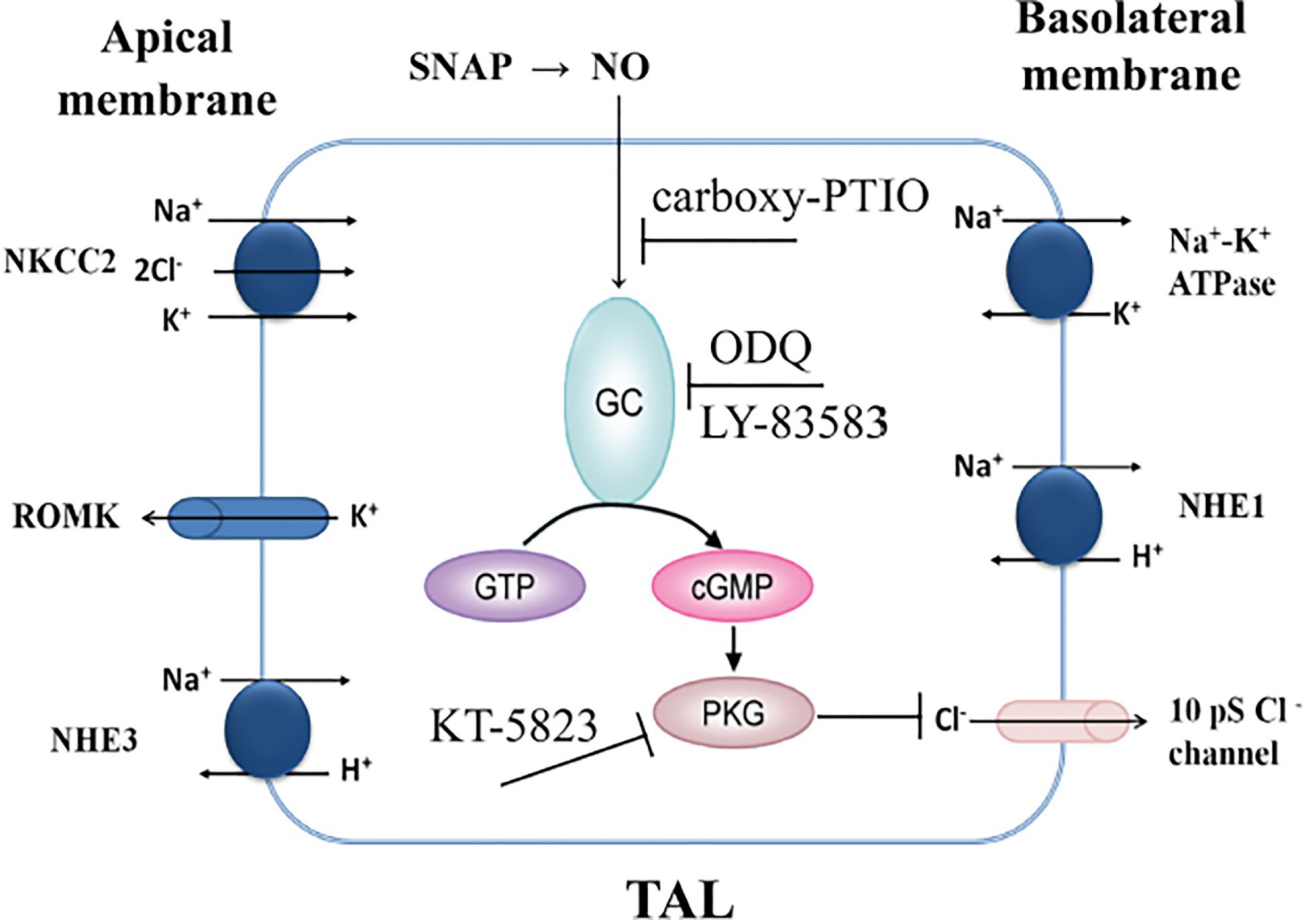
Signaling cascade for Inhibitory effect of SNAP on the 10-pS Cl^-^ channel in TAL. Carboxy-PTIO is a NO scavenger, ODQ or LY-83583 blocks activity of GC, KT-5823 inhibits PKG.

Historically, the sodium absorption pathways have been studied much more than that of the chloride pathways. However, as a matter of fact, the absorption of both ions is mutually dependent with each other, which is critical for regulation of extracellular fluid volume as well as blood pressure [10]. In the present study, we found that SNAP inhibited this basolateral 10-pS Cl^-^ channel in the TAL in a dose-dependent manner with an IC50 value of 6.6 μM. Furthermore, we showed that the inhibitory effect of SNAP on the channel was through activation of the cGMP-dependent PKG pathway but not through the cGMP-stimulated PDE2 pathway. A model of the signaling pathway for the inhibitory effect of SNAP on the basolateral 10-pS Cl^-^ channel in TAL is presented in Fig 7. NO is released exogenously by SNAP (NO donor), diffuses across the plasma membrane. In the cytoplasm, NO reacts with GC, and stimulates the production of cGMP. This intracellular messenger in turn activates PKG, and inhibits the basolateral 10-pS Cl channels in TAL. Increase in activity of this channel in the TAL has been shown to be associated with hypertension [13,35,36]. It is reasonable to assume that inhibitors of the 10-pS Cl-channel, such as SNAP, may have the potential of being developed as anti-hypertension agents.

## Supporting information

S1 File Experimental data for Fig 1B. (XLS)

S2 File Experimental data for Fig 1C. (XLS)

S3 File Experimental data for Fig 2B. (XLS)

S4 File Experimental data for Fig 3B. (XLS)

S5 File Experimental data for Fig 4B. (XLS)

S6 File Experimental data for Fig 5B. (XLS)

S7 File Experimental data for Fig 6B. (XLS)
